# Three-dimensional gait analysis for assessing dynamic ankle spasticity after stroke

**DOI:** 10.1186/s12984-026-01968-x

**Published:** 2026-04-02

**Authors:** Zengqiang Ouyang, Yifan Wang, Shanshan Xiong, Rui Yang, Yue Wang

**Affiliations:** 1 Department of Neurology and Neurological Rehabilitation, Shanghai YangZhi Rehabilitation Hospital (Shanghai Sunshine Rehabilitation Center), School of Medicine, Tongji University, 2209 Guangxing Road, Songjiang District, Shanghai, 201619 China; 2 Kinesiology Lab, Shanghai YangZhi Rehabilitation Hospital (Shanghai Sunshine Rehabilitation Center), School of Medicine, Tongji University, Shanghai, 201619 China

**Keywords:** Dynamic spasticity, Motion analysis, Walking, Ankle, Stroke

## Abstract

**Background:**

Dynamic ankle spasticity during walking is a common complication after stroke. It remains unclear which clinical indicators predict dynamic spasticity and whether three-dimensional (3D) gait parameters can reflect its severity.

**Methods:**

3D gait analysis data were obtained from 42 patients with dynamic spasticity and a 1:1 propensity score-matched control group. Spasticity was assessed using the Modified Tardieu Scale (MTS). Dynamic kinematic and spatiotemporal gait indicators were analyzed. Spearman correlation analysis was performed to examine associations between gait parameters and MTS scores. A binary logistic regression model (Model 1) was constructed to identify factors associated with dynamic spasticity using gait-related physical assessments and ankle kinematics, with stepwise backward elimination applied. For severity evaluation, an ordinal logistic regression model (Model 2) was built using the MTS X score as the dependent variable.

**Results:**

Compared to controls, the spasticity group showed slower gait speed, shorter step length, and longer double support time. In Model 1, reduced ankle plantarflexion angle and weaker dorsiflexor strength were independently associated with the presence of dynamic spasticity. In Model 2, prolonged double support time on the affected side was significantly associated with dynamic spasticity severity.

**Conclusion:**

Reduced plantarflexion angle and dorsiflexor weakness are key clinical factors associated with walking-related dynamic spasticity, and prolonged double support time may serve as a quantifiable indicator of its severity. These findings support the potential value of 3D gait analysis in identifying and characterizing dynamic spasticity after stroke.

**Supplementary Information:**

The online version contains supplementary material available at 10.1186/s12984-026-01968-x.

## Background

Spasticity is a common complication in stroke survivors with hemiparesis [1, 2], characterized primarily by a velocity-dependent increase in stretch reflex excitability [3]. It is widely recognized as a typical manifestation of the upper motor neuron syndrome [4]. Among all affected joints, ankle spasticity is one of the most significant factors impacting gait and dynamic balance in patients [5]. It often leads to various ankle-foot deformities (equinus, varus, and equinovarus postures), with spastic equinovarus being the most frequently observed presentation [6]. During walking, the severity and manifestation of ankle spasticity vary dynamically across different phases of the gait cycle [7]. Such dynamic ankle spasticity can profoundly impair patients’ walking ability and functional independence in daily life [8].

Currently, clinicians lack standardized criteria for determining when dynamic spasticity warrants intervention. In addition, there is still no reliable method for accurately assessing ankle spasticity during walking [9–13]. According to patient-reported surveys, some individuals describe involuntary muscle tightening or jerking sensations in their lower limbs during specific phases of gait (e.g., initial contact or toe-off), with symptoms fluctuating throughout the gait cycle. These observations suggest the need for more objective and quantitative assessment tools to capture such walking-related manifestations of dynamic spasticity [9, 14, 15].

Currently, commonly used clinical assessment tools include the Modified Ashworth Scale (MAS) and the Modified Tardieu Scale (MTS). Among them, the MTS offers higher sensitivity because it differentiates between the static and dynamic components of spasticity through velocity-dependent stretch testing [16]. However, both scales are performed under passive conditions. This means they cannot capture spasticity that appears only during active and functional tasks, such as walking. As a result, these tools may underestimate the true extent of spasticity during dynamic movements [17]. Their ability to identify dynamic spasticity in clinical practice is therefore limited.

In recent years, three-dimensional (3D) gait analysis has become a comprehensive evaluation technique that integrates spatial trajectories, joint kinematics, and spatiotemporal parameters. This approach provides a strong basis for quantifying dynamic spasticity during walking [8, 18]. Previous studies have shown that targeted interventions for spasticity in the soleus or tibialis posterior muscles can lead to significant improvements in ankle angles at initial contact and peak dorsiflexion [7]. These changes are often accompanied by faster gait speed and better balance [19]. In addition, the severity of hamstring spasticity assessed using the MAS and MTS has been significantly associated with gait-based markers, such as knee extension angles and hamstring lengthening velocity [20]. Despite these findings, no systematic research has examined whether dynamic ankle spasticity can be predicted using 3D gait characteristics or whether its severity can be quantitatively assessed through gait parameters.

This study addresses an important clinical gap, as current spasticity assessments are largely based on static examinations and therefore cannot fully capture the dynamic ankle spasticity that occurs during walking. To address this issue, the first objective (Model 1) was to identify which routinely used gait-related physical assessment variables are associated with the presence of walking-related dynamic spasticity. These findings may help clinicians and therapists anticipate the likelihood of dynamic spasticity during routine bedside assessment, even before 3D gait analysis is performed. The second objective (Model 2) was to determine which spatiotemporal and kinematic parameters obtained from 3D gait analysis can reflect the severity of dynamic spasticity during real walking in patients with stroke. To our knowledge, this is the first study to integrate the MTS with 3D gait analysis within a matched design, with the aim of identifying objective, movement-based markers that support early detection and guide individualized rehabilitation planning.

## Methods

### Experimental subjects

Consecutive patients diagnosed with stroke in Shanghai YangZhi Rehabilitation Hospital (Shanghai Sunshine Rehabilitation Center) Affiliated to Tongji University, from October 2022 to December 2024, were enrolled. The inclusion criteria were: (1) Aged ≥ 18 years; (2) Diagnosed with unilateral hemiparesis resulting from a stroke; (3) Undergone a standardized MTS assessment conducted by the research team in the gait laboratory before motion capture; (4) Able to walk independently without assistive devices and complete the 3D gait assessment protocol. Exclusion criteria were: (1) Bilateral limb involvement; (2) Multiple brain injuries; (3) Prior surgery involving the lower limb joints, tendons, or related structures; (4) Implanted devices such as electrical or magnetic stimulators; (5) Botulinum toxin injections within the previous six months.

The present study was approved by the Ethics Committee of Shanghai YangZhi Rehabilitation Hospital (Shanghai Sunshine Rehabilitation Center) (reference numbers: 2023-033-CR-01 and 2024-073).

### Gait-related physical evaluation

#### Assessment of spasticity

Spasticity was assessed using the MTS with participants positioned in a supine posture. The MTS evaluates three key components: the velocity of stretch, the angle-based response to stretch, and the quality of the muscle response (Tardieu X score). The assessment began with a slow passive movement (designated as V1) through the full available range of motion. The angle reached at the end of this movement was measured using a standard universal goniometer and recorded as R2, representing the passive range of motion. Then, a rapid passive stretch (designated as V3) was applied in the same direction. If a spastic response (e.g., a catch or clonus) occurred during the fast stretch, the angle at which it was first elicited was recorded as R1. The difference between R2 and R1 (R2-R1) is referred to as the “Y value” in this study. The quality of the spastic response during V3 was scored using the X score, ranging from 0 (no resistance) to 4 (severe resistance with clonus), based on standardized criteria. This qualitative assessment reflects the intensity and abruptness of muscle resistance during rapid stretch [21].

#### Assessment of muscle strength

Manual muscle testing (MMT) was used to assess ankle muscle strength. Muscle strength was graded using a 6-point scale (0–5): Grade 0 (no muscle contraction), Grade 1 (palpable or visible muscle contraction), Grade 2 (full range of motion in a gravity-eliminated position), Grade 3 (full range of motion against gravity), Grade 4 (movement against moderate resistance), and Grade 5 (movement against maximum resistance, considered normal strength). MMT assesses the function and strength of muscles by observing their ability to perform specific movements against the forces of gravity and manual resistance [22].

#### Measurement of the passive range of motion of the ankle joint

The subject lay supine with the knee fully extended and the tested ankle hanging freely over the edge of the table. The subtalar joint was kept in a neutral position, confirmed by a second examiner. One examiner stabilized the lower leg and applied a slow, steady force to the plantar surface of the foot until firm resistance was felt. The ankle angle was then measured using a standard goniometer [23]. A reduced passive dorsiflexion angle indicates tightness, shortening, or possible spasticity of the posterior calf muscles (gastrocnemius–soleus complex), whereas a reduced passive plantarflexion angle suggests tightness or reduced extensibility of the ankle dorsiflexor muscles.

#### Popliteal angle test

The subject lay supine with the hip flexed to 90°. The examiner stabilized the hip and slowly extended the knee until resistance was felt and no further extension was possible. The popliteal angle was then measured with a standard goniometer [24]. An increased popliteal angle indicates reduced hamstring extensibility and may suggest hamstring tightness or spasticity.

#### The Ely test

The Ely test was performed with the subject in a prone position and both lower limbs extended. The examiner passively flexed the knee to bring the heel toward the buttocks. If the ipsilateral hip lifted off the table, the test was considered positive [25]. A positive Ely test indicates reduced extensibility or tightness of the rectus femoris and may also suggest rectus femoris spasticity.

#### The circumference of the lower leg

For lower leg circumference measurement, the subject was asked to keep the lower leg relaxed. The midportion of the lower leg, specifically the most prominent part of the calf, was selected. A flexible measuring tape was wrapped around this area, ensuring that the tape adheres closely to the skin without being excessively tight. The reading was then recorded.

### Devices and experimental procedure

An eight-camera infrared motion capture system (MX T40-S, Vicon, Oxford, UK) was used to collect kinematic 3D data at a sampling frequency of 100 Hz. In addition, two video cameras (JVC) were positioned to record participants’ walking from the front and the side. The IOR-Lower Limb-26 model was employed to reconstruct lower limb joints and rigid body segments. All trials were recorded and processed using the Vicon Nexus software (version 1.8.6, Vicon, Oxford, UK).

**Fig. 1:**
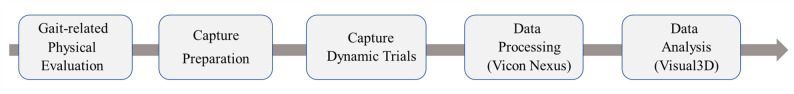
The procedure of the experiment. Following gait-related physical assessment, 3D gait data capture, processing, and analysis were performed sequentially

Participants were first asked to lie down on a treatment table. Physical therapists then performed gait-related physical assessments, including spasticity evaluations, passive ankle range of motion, muscle strength testing, the popliteal angle test, the Ely test, and the lower leg circumference measurement. Demographic and clinical information, including gender, age, age at onset, height, and weight, was collected and documented. Following the assessment, participants stood in a designated area, and reflective markers for the IOR-Lower-Limb-26 model were placed on specific anatomical landmarks by the physical therapists. Static and dynamic trials were then recorded. The experimental procedures were carried out by two professionally trained physical therapists (Yifan Wang and Zengqiang Ouyang), and all outcomes were verified by a chief physician (Yue Wang). The experimental procedure is illustrated in Fig. 1.

### Data capture

**Fig. 2:**
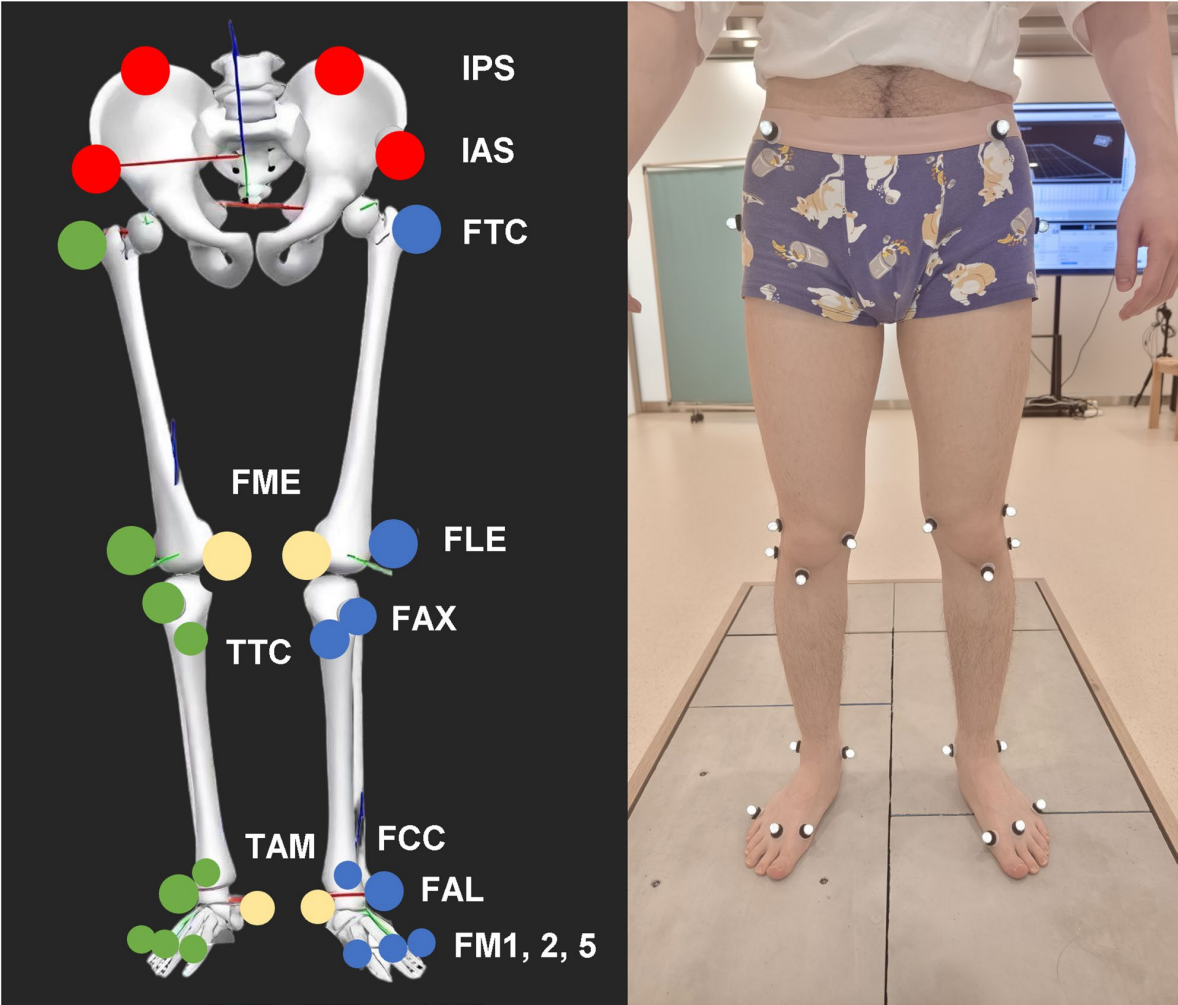
Reflective markers for the IOR lower limb model. IAS: anterior superior iliac spine; IPS: posterior superior iliac spine; FTC: femoral greater trochanter; FLE: lateral femoral epicondyles; FME: medial femoral epicondyles; TTC: tibial tuberosity; FAX: apex of the fibular styloid process; TAM: tibial apex of medial malleolus; FAL: lateral malleolus; FCC: achilles tendon insertion on the calcaneus; FM1: the dorsal aspects of the first metatarsal head; FM2: the dorsal aspects of the second metatarsal head; FM5: the dorsal aspects of the fifth metatarsal head

Participants were asked to change into shorts and a shirt that exposed the abdomen and to stand barefoot. Reflective markers for the IOR lower limb model (26 markers) were then placed on the skin by physical therapists at specific anatomical landmarks, including the anterior superior iliac spine (IAS), posterior superior iliac spine (IPS), femoral greater trochanter (FTC), lateral and medial femoral epicondyles (FLE, FME), tibial tuberosity (TTC), the apex of the fibular styloid process (FAX), tibial apex of medial malleolus (TAM), lateral malleolus (FAL), Achilles tendon insertion on the calcaneus (FCC), and the dorsal aspects of the first, second, and fifth metatarsal heads (FM1, FM2, FM5), bilaterally (Fig. 2) [26].

We first acquired a static trial to record the positions of the retro-reflective markers, which were subsequently used to instantiate an anatomical skeletal model in Visual3D. Dynamic walking trials were then collected. In these trials, participants walked along a 6-meter walkway at a self-selected comfortable pace without assistive devices, while physical therapists provided guarding to prevent falls. Each valid trial included at least two consecutive complete gait cycles, with 4–6 valid trials collected per participant [27].

### Data processing and analysis

In this study, the 3D gait analysis focused on two conventional domains: joint angles and spatiotemporal parameters. This decision was primarily based on clinical feasibility and the accessibility of data collection. Kinematic and spatiotemporal parameters are among the most common and stable outputs in 3D gait laboratories, with good repeatability and relatively low technical demands [28–30].

Vicon Nexus 1.8.6 was used to create the virtual lab environment and to capture and reconstruct the complete motion capture trials. Physical therapists manually labeled the reflective markers and interpolated missing trajectories by filling data gaps. In dynamic trials, the low-pass filter (6 Hz cut-off, fourth-order, zero-lag) was applied to the raw marker trajectories during the gap-filling process. This filtering step aims to reduce high-frequency noise caused by marker vibration during walking. The processed trials were then exported to Visual3D software (Visual3D x64 Professional v2020.07.1, C-Motion Inc., Germantown, MD, USA), where the data were further filtered using a low-pass filter at 6 Hz to reduce noise artifacts. Visual3D was subsequently used to calculate kinematic variables (including the ankle angle at initial contact, initial swing, maximum dorsiflexion, and maximum plantarflexion) as well as spatial and temporal gait parameters (speed, cadence, step length, step width, stance phase, swing phase, and gait cycle) (Supplementary Fig. 1) [31]. Values were extracted for both the affected and unaffected sides.

#### Kinematic variables

*Ankle angle* is automatically calculated in the Visual3D software. It refers to the angle formed between the shank and the foot. Both segments are defined by their proximal and distal parts. For the shank segment, the proximal joint center of the proximal part is determined by trajectories FLE and FME, while the distal part is determined by trajectories FAL and TAM. With respect to the foot, the joint center of the proximal part is determined by trajectories FAL and TAM, and the distal part is determined by trajectories FM5 and FM1. Thus, the ankle joint angle is the angle between the line connecting the proximal and distal joint centers of the shank and the analogous line for the foot. The key gait events used to extract ankle angle characteristics are illustrated in Fig. 2, including the initial contact ankle angle, toe-off ankle angle, maximum ankle dorsiflexion angle, and maximum ankle plantarflexion angle.

*Initial contact ankle angle* refers to the ankle angle measured at the moment the foot first contacts the ground.

*Toe-off ankle angle* refers to the ankle angle measured at the moment the foot leaves the ground.

*Maximum dorsiflexion angle and maximum plantarflexion angle* represent the peak positive and negative values of the ankle angle, respectively, throughout the gait cycle.

#### Spatial and temporal gait parameters

*Speed* is the rate at which a person walks and is calculated using the following formula:

$$\:Speed=\frac{Distance}{Time}$$

*Cadence*, defined as the number of steps taken per minute during walking, is calculated using the following formula:

$$\:Cadence=\frac{Steps}{Time}$$

In this study, all cadence values were automatically generated by the Visual3D software, which computes the number of steps per unit time based on both the affected and unaffected sides.

*Step length* is the distance between the point of initial contact of one foot and the point of initial contact of the opposite foot.

*Step length (% height)* is a normalized gait parameter that expresses step length as a percentage of a person’s body height. It is calculated using the following formula:

$$\:Step\:length\:\left(\%\:height\right)\:=\frac{Step\:length}{Height}\:\times\:\:100\%$$

*Step width* is the mediolateral (horizontal) distance between the heel centers of two consecutive foot contacts, typically measured during walking.

*Gait cycle* refers to the period from the initial contact of one foot with the ground to the next initial contact of the same foot. It consists of two main phases: the stance phase and the swing phase. The stance phase is the duration during which the foot is in contact with the ground. The swing phase is the duration during which the foot is off the ground and swinging forward. The gait cycle time is the sum of the stance phase time and swing phase time (Supplementary Fig. 1). The percentage of each phase in the gait cycle is typically calculated using the following formula:

$$ \begin{gathered} \,Stance/Swing\,phase\,ratio\,\% \hfill \\ = \frac{{Stance/Swing\,phase\,time}}{{Gait\,cycle\,time}}\, \times \,\,100\% \hfill \\ \end{gathered} $$

*Double support* is the period during the gait cycle when both feet are in contact with the ground. It occurs twice in each gait cycle and includes two distinct phases:

*Initial double support* is the period when the reference foot makes initial contact with the ground and ends when the contralateral foot lifts off.

**Fig. 3:**
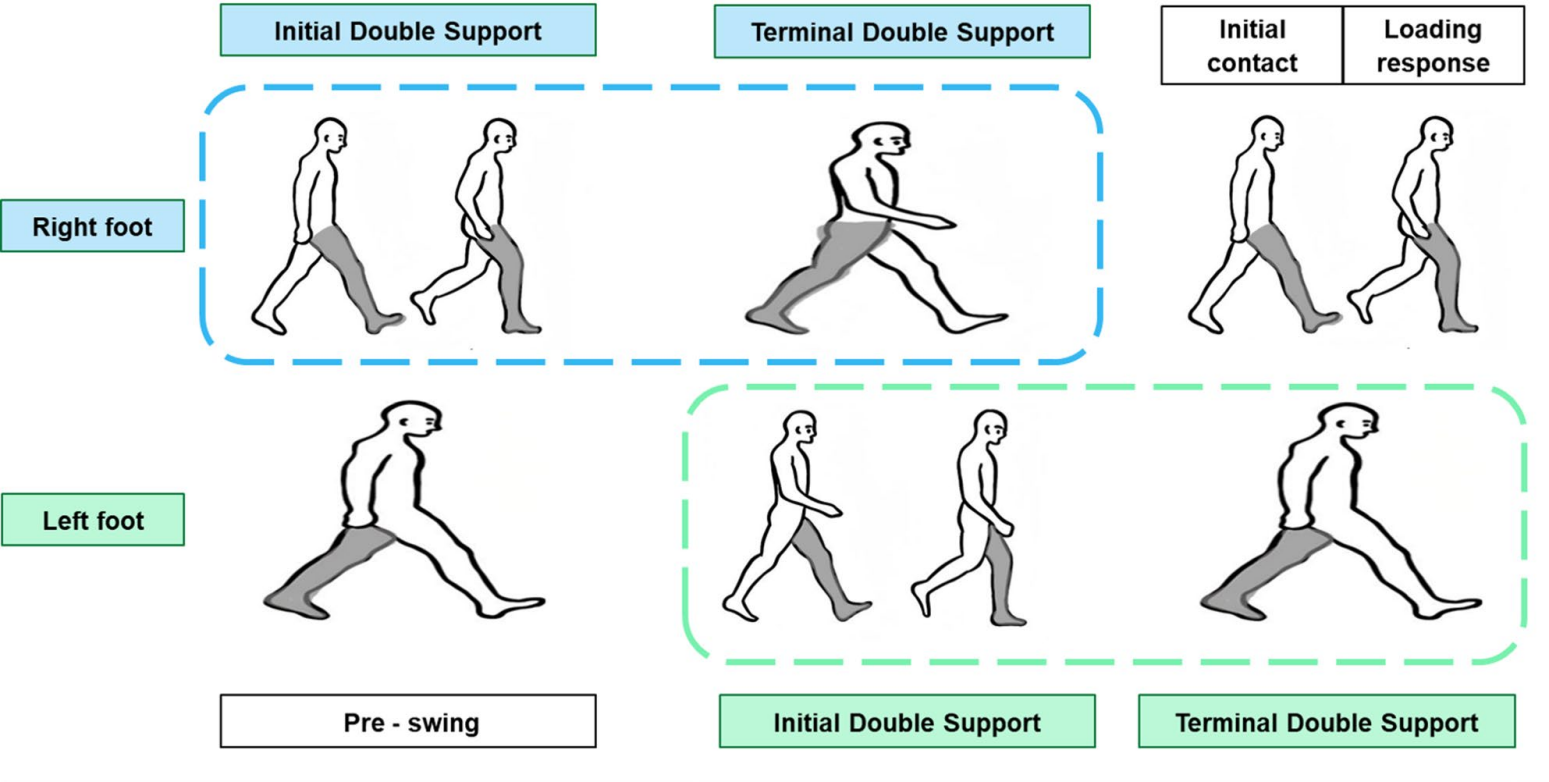
Initial and terminal double support phases in the gait cycle of the right and left lower limbs. The blue dashed box represents the double support phases (initial and terminal) within one complete gait cycle of the right foot, and the green dashed box represents the double support phases within one complete gait cycle of the left foot. The right and left limbs are analyzed as separate reference limbs, each with its own distinct gait cycle. As such, the duration of double support phases can differ between the affected and unaffected sides. Initial double support is defined as the phase when the reference limb progresses from initial contact to loading response and the contralateral limb is at pre-swing, while terminal double support is defined as the phase when the reference limb is at pre-swing and the contralateral limb progresses from initial contact to loading response

*Terminal double support* is the period when the contralateral foot makes contact with the ground again and ends when the reference foot lifts off (Fig. 3).

### Statistical analysis

To reduce confounding and strengthen causal inference by balancing key covariates between groups, we performed propensity score matching (PSM). Propensity scores were estimated using logistic regression with age and sex included as covariates, as these variables are among the most commonly used and clinically relevant factors in PSM. The ankle spasticity group initially included 44 patients, whereas the non-spastic group included 118 patients. Matching was performed using 1:1 nearest-neighbor matching without replacement and a caliper of 0.2 standard deviations of the logit of the propensity score, resulting in 42 well-matched pairs with balanced covariates (standardized mean differences < 0.1 for all matched variables).

The normality of continuous variables was assessed using the Shapiro-Wilk test. Normally distributed variables were presented as the mean ± standard deviation (SD), while non-normally distributed variables were expressed as the median with interquartile range (IQR). Categorical variables were summarized as frequencies and percentages. Between-group comparisons were performed using independent samples t-tests for normally distributed data, Mann-Whitney U tests for non-normally distributed data, and chi-square tests for categorical variables. Variables that showed significant between-group differences were further examined using Spearman correlation to assess the strength of their association with MTS scores and to identify the most suitable candidates for multivariate modelling (r_s_ = 0.10–0.29 as weak, 0.30–0.49 as moderate, and ≥ 0.50 as strong).

For multivariate analysis, the following variables were included in a binary logistic regression model (Model 1): duration, dorsiflexion angle (affected side), plantarflexion angle (affected side), and muscle strength of dorsiflexors and plantarflexors (affected side). A stepwise backward elimination approach (Likelihood Ratio method) was applied, resulting in a final model retaining four variables that remained associated with the outcome. The likelihood of dynamic spasticity during walking after stroke was estimated using odds ratios (ORs) and 95% confidence intervals (CIs).

To further identify which 3D gait parameters reflect the severity of dynamic spasticity in patients already exhibiting spasticity, an ordinal logistic regression model (Model 2) was constructed using the Tardieu X score of the MTS assessment as the dependent variable. As the X score is an ordinal categorical variable reflecting velocity-dependent resistance, the proportional odds model with a logit link function was applied. Only participants with a Tardieu X score greater than 0 were included in this analysis. Before modeling, Spearman correlation analysis was performed to identify 3D gait variables significantly associated with the X score (*p < 0.05*). To address multicollinearity, variables with high correlations (r_s_ > 0.7) or those considered clinically collinear were excluded, while variables with stronger clinical relevance were prioritized and retained. A stepwise selection procedure was used for model building. Model fit and the parallel lines assumption were assessed to ensure the validity of the ordinal logistic regression.

## Results

**Fig. 4:**
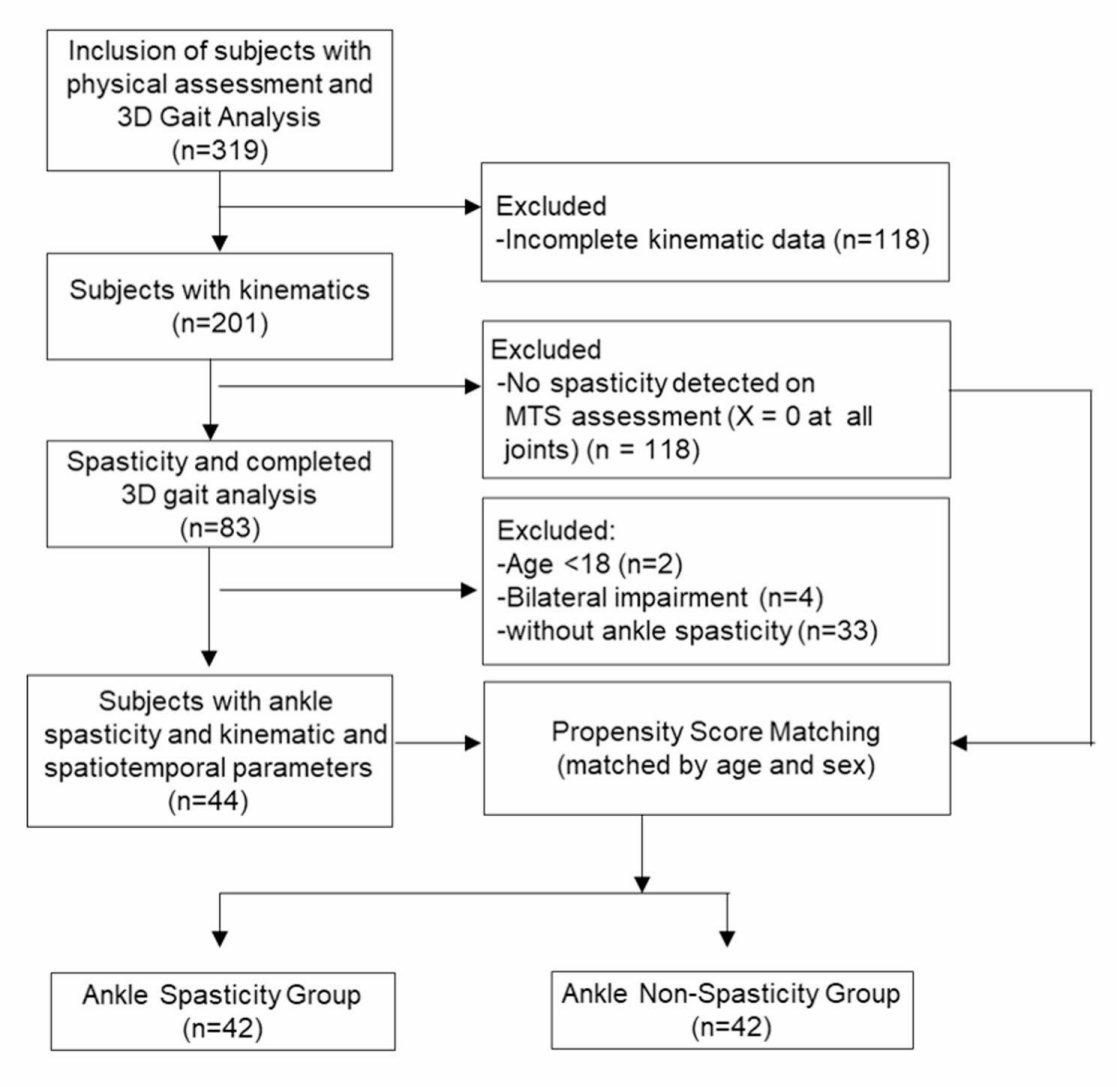
Flowchart of participant enrollment and propensity score matching. MTS: Modified Tardieu Scale

This was a propensity score-matched case-control study. A total of 319 cases were screened, and 44 cases were identified based on the inclusion and exclusion criteria, followed by PSM. Ultimately, a total of 42 stroke patients with ankle spasticity, identified using the MTS, were successfully matched 1:1 with 42 non-spastic stroke patients based on age and sex using PSM (Fig. 4).

### Demographic and physical assessment characteristics

Significant differences were observed between the spasticity and non-spasticity groups in disease duration, with a median duration of 4.0 (2.0–6.0) months. No significant differences were found between the two groups for age, gender, weight, height, and BMI. The mean age of participants was 53.5 ± 15.2 years, and the sample was predominantly male (65.5%) (Table 1, Supplementary Fig. 2).

**Table 1:** Demographic and physical assessment characteristics

	Total	Spasticity (42)	Non-spasticity (42)	*p*-value
Age (years)	53.5 ± 15.2	52.2 ± 15.6	54.9 ± 14.8	0.42
Male (n, %)	55 (65.5%)	29 (69.0%)	26 (61.9%)	0.49
Duration (months)	4.0 (2.0–6.0)	4.0 (2.0–8.0)	3.5 (0.9-6.0)	0.03*
Weight (kg)	67.1 ± 13.5	68.2 ± 14.4	65.9 ± 12.5	0.43
Height (cm)	166.5 ± 8.1	167.3 ± 9.4	165.6 ± 6.4	0.33
BMI (kg/m^2^)	23.9 (21.6–25.9)	23.6 (22.2–25.9)	24.1 (20.9–26.0)	0.87
Popliteal angle (unaffected, ˚)	49.0 (40.0–55.0)	50.0 (43.8–55.0)	44.5 (35.0–57.0)	0.26
Popliteal angle (affected, ˚)	50.0 (40.0–60.0)	56.0 (44.8–65.8)	44.5 (40.0–55.0)	0.007**
Dorsiflexion angle (unaffected, ˚)	5.0 (0.0–10.0)	5.0 (2.0–10.0)	3.5 (-1.52–6.0)	0.04*
Dorsiflexion angle (affected, ˚)	– 5 (– 12.8– 0.0)	– 8.0 (– 15.0 – – 4.8)	0.0 (– 6.3–3.5)	< 0.001***
Plantarflexion angle (unaffected, ˚)	52.0 (48.0–55.0)	50.0 (45.0–55.0)	55.0 (50.0–55.0)	0.15
Plantarflexion angle (affected, ˚)	48.7 ± 6.7	47.19 ± 6.2	50.2 ± 6.9	0.04*
Dorsiflexor strength (affected)	3 (1–5)	1 (0–4)	5 (3–5)	< 0.001***
Plantarflexor strength (affected)	5 (4–5)	4 (3–5)	5 (4–5)	< 0.001***
Calf circumference (unaffected, cm)	35.1 ± 3.6	35.5 ± 3.3	34.6 ± 3.8	0.26
Calf circumference (affected, cm)	34.7 ± 3.3	34.8 ± 2.9	34.5 ± 3.8	0.67
Ely test positive (unaffected, n, %)	8 (9.5%)	3 (7.1%)	5 (11.9%)	0.46
Ely test positive (affected, n, %)	13 (15.5%)	7 (16.7%)	6 (14.3%)	0.76
Tardieu X		3 (3–4)		
Tardieu Y (˚)		13.8 (9.5–19.0)		

*p < 0.05, **p < 0.01, ***p < 0.001. Data are presented as mean ± standard deviation for normally distributed variables and as median (interquartile range) for non-normally distributed variables. Categorical variables are shown as number (percentage). BMI: body mass index, Bold values indicate statistical significance (p < 0.05)

Significant differences in physical metrics were observed for the affected‑side popliteal angle, dorsiflexion angle, plantarflexion angle, dorsiflexor strength, and plantarflexor strength, as well as the unaffected‑side dorsiflexion angle. No statistically significant differences were observed in calf circumference or Ely test results between groups. In the spasticity group, the Tardieu X score was 3 (3–4), and the Y value was 13.8° (9.5–19.0°) (Table 1, Supplementary Fig. 2).

### Kinematic variables

Significant differences were found in the affected side initial contact ankle angle, affected side maximum dorsiflexion angle, and unaffected side initial contact ankle angle. The unaffected side toe-off ankle angle tended to be lower in the spasticity group. No significant differences in the remaining ankle angles were observed between the two groups (Table 2, Supplementary Fig. 3).

**Table 2:** Kinematic variables and spatiotemporal parameters

	Total	Spasticity (42)	Non-spasticity (42)	*p*-value
Initial contact ankle angle (unaffected, °)	– 1.0 ± 5.9	0.3 ± 6.9	– 2.4 ± 4.4	0.04*
Toe-off ankle angle (unaffected, °)	– 2.2 ± 5.8	– 1.1 ± 6.4	– 3.3 ± 4.9	0.07
Max dorsiflexion angle (unaffected, °)	15.4 ± 4.6	15.5 ± 4.5	15.2 ± 4.6	0.79
Max plantarflexion angle (unaffected, °)	– 9.0 ± 6.3	– 8.4 ± 6.5	– 9.6 ± 6.1	0.39
Initial contact ankle angle (affected, °)	– 4.8 ± 5.4	– 6.0 ± 6.0	– 3.6 ± 4.6	0.04*
Toe-off ankle angle (affected, °)	– 1.3 ± 5.6	– 0.9 ± 6.7	– 1.8 ± 4.2	0.49
Max dorsiflexion angle (affected, °)	11.1 ± 5.9	9.2 ± 6.8	13.0 ± 3.9	0.002**
Max plantarflexion angle (affected, °)	– 10.0 ± 6.2	– 10.8 ± 6.5	– 9.2 ± 5.8	0.25
Speed (cm/s)	29.0 (15.2–52.2)	21.3 (10.9–44.4)	40.9 (20.2–73.6)	< 0.001***
Cadence (unaffected, steps/min)	78.7 (50.1–93.1)	70.0 (47.7–91.3)	82.0 (51.0–103.0)	0.10
Cadence (affected, steps/min)	64.0 (36.0–87.5)	51.1(32.2–72.5)	82.9 (47.4–100.6)	< 0.001***
Step width (cm)	16.0 ± 4.5	17.2 ± 4.6	14.7 ± 1.0	0.01*
Step length (unaffected, cm)	28.6 ± 13.53	24.8 ± 10.9	32.4 ± 14.9	0.009**
Step length (affected, cm)	32.7 ± 11.0	29.5 ± 9.2	35.8 ± 11.9	0.007**
Step length (unaffected, % height)	17.2 ± 8.2	14.8 ± 6.4	19.6 ± 9.1	0.007**
Step length (affected, % height)	18.2 (14.9–22.5)	17.0 (13.9–21.6)	20.4 (16.7–29.3)	0.01*
Stance phase (unaffected, s)	1.3 (0.9–2.40)	1.7 (1.1–2.8)	1.1 (0.7–1.9)	< 0.001***
Stance phase (affected, s)	1.2 (0.9–2.1)	1.5 (0.9–2.5)	1.1 (0.8–1.9)	0.005**
Double support (unaffected, s)	0.4 (0.2–0.9)	0.6 (0.3–1.4)	0.3 (0.2–0.6)	< 0.001***
Double support (affected, s)	0.4 (0.2–0.7)	0.5 (0.3–0.8)	0.3 (0.2–0.7)	0.02*
Stance phase (% cycle, unaffected)	78.5 (68.4–86.6)	83.8 (73.6–89.1)	74.0 (64.4–81.7)	< 0.001***
Stance phase (% cycle, affected)	69.7 (63.7–78.7)	71.1 (65.4–78.7)	68.3 (63.0-79.5)	0.28
Double support (% cycle, unaffected)	22.1 (16.3–34.7)	25.6 (18.1–43.0)	18.8 (14.8–28.5)	0.004**
Double support (% cycle, affected)	21.6 (15.4–29.1)	20.9(17.8–30.7)	21.7 (14.0-27.3)	0.35
Swing phase (unaffected, s)	0.4 ± 0.1	0.4 ± 0.1	0.4 ± 0.1	0.10
Swing phase (affected, s)	0.5 ± 0.2	0.7 ± 0.2	0.5 ± 0.1	< 0.001***
Swing phase (% cycle, unaffected)	21.5 (13.4–31.6)	16.2 (10.5–26.4)	26.0 (18.3–35.6)	< 0.001***
Swing phase (% cycle, affected)	30.3 (21.3–36.3)	28.9 (21.3–34.6)	31.7 (20.5–37.0)	0.28
Gait cycle duration (unaffected, s)	1.7 (1.4–2.8)	2.1 (1.5-3.0)	1.5 (1.2–2.3)	< 0.001***
Gait cycle duration (affected, s)	1.7 (1.3–2.8)	2.1 (1.5-3.0)	1.4 (1.2–2.3)	0.001**

*p < 0.05, **p < 0.01, ***p < 0.001. Data are presented as mean ± standard deviation for normally distributed variables and as median (interquartile range) for non-normally distributed variables. Categorical variables are shown as number (percentage), Bold values indicate statistical significance (p < 0.05)

### Spatiotemporal parameters

Compared with the non-spasticity group, the spasticity group exhibited multiple abnormalities in spatiotemporal gait parameters. Patients in the spasticity group walked significantly slower, with lower cadence, wider step width, and shorter step lengths on both the affected and unaffected sides. Step length normalized to height was also significantly lower in the spasticity group for both limbs (Table 2, Supplementary Fig. 4).

In terms of temporal parameters, the spasticity group demonstrated significantly prolonged stance phase and double support time on both the affected and unaffected sides. They also spent a greater proportion of the gait cycle in stance and double support. Additionally, the spasticity group had a significantly longer swing phase on the affected side, whereas the non-spasticity group spent a greater percentage of the gait cycle in the swing phase on the unaffected side. Gait cycle duration was significantly prolonged in the spasticity group for both the affected and unaffected sides (Table 2, Supplementary Fig. 4).

### Correlation analysis

Further correlation analyses were performed between the variables that showed significant differences in Tables 1 and 2 and the X and Y values of the MTS within the spasticity group.

Correlation analyses within the spasticity group revealed that most physical assessment variables were not significantly associated with either the Tardieu Y or X values. However, a moderate negative correlation was observed between affected side dorsiflexor strength and the X score, suggesting that greater spasticity severity was associated with weaker dorsiflexor strength. Similarly, a significant negative correlation was found between plantarflexor strength and the X score. In addition, the dorsiflexion angle of the affected ankle was significantly correlated with the X score. No significant associations were found between disease duration or popliteal angle and either the Y or X score (Table SM 1).

Correlation analyses were conducted between kinematic and spatiotemporal gait parameters and MTS scores (X and Y) within the spasticity group. The X score demonstrated significant correlations with several gait parameters. Specifically, higher X scores were associated with increased stance phase duration on both the affected side and the unaffected side, longer double support time on the affected side, prolonged gait cycle duration on both sides, and reduced step width. In addition, both the initial contact ankle angle on the affected side and swing phase duration were significantly correlated with the X score.

**Fig. 5:**
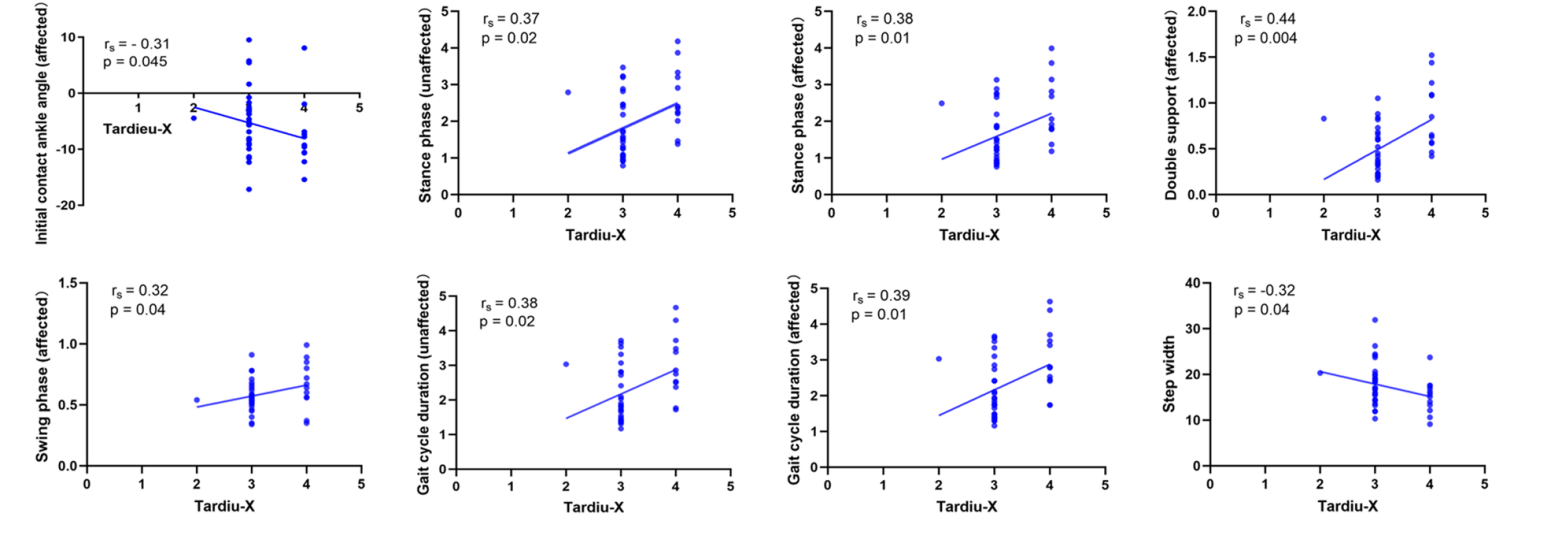
Correlation between Tardieu X scores and kinematic and spatiotemporal gait parameters in the spasticity group

In contrast, the Y score showed fewer associations. A significant negative correlation was found between swing phase duration on the affected side and the Y score, and a positive correlation was observed between the unaffected initial contact ankle angle and the Y score. No other gait parameters showed significant relationships with either score (Table SM 2, Fig. 5).

### Multivariate analysis of dynamic spasticity during walking

*Model 1* aimed to identify factors associated with spasticity by performing multivariate analysis based on demographic characteristics and physical gait assessments obtained prior to gait analysis, to differentiate between patients with and without spasticity.

To identify independent factors associated with dynamic spasticity during walking after stroke, a multivariate logistic regression analysis was conducted using a backward stepwise elimination method based on likelihood ratio testing (Backward: LR). Five variables that showed statistical significance in univariate and correlation analyses—including disease duration, dorsiflexion angle, plantarflexion angle, dorsiflexor strength, and plantarflexor strength (all on the affected side)—were initially entered into model 1. After stepwise elimination, the final model retained four variables. Among these, a reduced plantarflexion angle (adjusted OR = 0.909, 95% CI: 0.833–0.992, *p* = 0.032) and decreased dorsiflexor strength (adjusted OR = 0.679, 95% CI: 0.501–0.921, *p* = 0.013) on the affected side remained significantly associated with dynamic spasticity. In contrast, disease duration and dorsiflexion angle were not statistically significant in the final model (Tables 3 and 5).

**Table 3:** Model 1: Estimating the likelihood of dynamic spasticity during walking after stroke

	OR (95%CI)	*p*-value	Adjusted OR (95%CI)	*p*-value
Duration (months)	1.13 (1.003–1.277)	0.044	1.12 (0.973–1.290)	0.115
Dorsiflexion angle (affected, ˚)	0.91 (0.865–0.965)	0.001	0.95 (0.888–1.009)	0.094
Plantarflexion angle (affected, ˚)	0.931 (0.869–0.997)	0.041	0.91 (0.833–0.992)	**0.032**
Dorsiflexor strength (affected)	0.60 (0.464–0.776)	< 0.001	0.68 (0.501–0.921)	**0.013**

Bold values indicate statistical significance (p< 0.05)

**Table 4:** Overview of the two regression models

Model	Objective	Sample size	Number of candidate variables	Candidate variables	Significant variables retained
Model 1	Identify physical assessment indicators predicting dynamic spasticity occurrence	84	4	• Duration (months) • Dorsiflexion angle (affected side, °) • Plantarflexion angle (affected side, °) • Dorsiflexor strength (affected side)	• Plantarflexion angle (affected side, °) • Dorsiflexor strength (affected side)
Model 2	Determine 3D gait parameters associated with dynamic spasticity severity	42	3	• Initial contact ankle angle (affected side, °) • Double support time (affected side, s) • Step width (cm)	• Double support time (affected side, s)

Model 1 identifies physical assessment indicators associated with the occurrence of dynamic spasticity. Model 2 determines 3D gait parameters related to the severity of dynamic spasticity. Candidate variables were entered into stepwise regression models, and only statistically significant predictors were retained in the final models

*Model 2* was constructed to investigate gait-related parameters associated with the severity of dynamic spasticity during walking in patients already exhibiting spasticity.

Only participants with an X score greater than 0 were included. The final analysis sample consisted of 42 individuals in the spasticity group. Before modeling, Spearman correlation analysis was performed to identify spatiotemporal and kinematic variables significantly associated with the X score (*p* < 0.05). To address multicollinearity, variables with high intercorrelation (r_s_ > 0.7) were excluded (Table SM 3), and those with stronger clinical relevance were retained. 3D gait parameters—initial contact ankle angle, double support time on the affected side, and step width—were included in the final model using a stepwise selection approach. Among them, double support time on the affected side showed a significant association with increased spasticity severity, suggesting that longer double support time reflects more severe motor impairment and poorer spasticity control. In contrast, initial contact angle and step width were not significantly associated with spasticity severity (Tables 4 and 5).

**Table 5:** Model 2: Functional gait indicators associated with the severity of dynamic ankle spasticity

	OR (95%CI)	*p*-value	Adjusted OR (95%CI)	*p*-value
Initial contact ankle angle (affected, °)	0.91 (0.799–1.030)	0.133	0.93 (0.809–1.076)	0.346
Double support (affected, s)	29.63 (2.662-336.974)	0.006	22.10 (1.877-258.688)	**0.014**
Step width (cm)	0.85 (0.720–1.010)	0.065	0.90 (0.747–1.082)	0.259

“OR” refers to the results from univariate ordinal logistic regression analysis

“Adjusted OR” refers to the results from the multivariable ordinal logistic regression model after covariate adjustment

Bold values indicate statistical significance (p< 0.05)

## Discussion

This study integrated clinical gait assessments with 3D gait analysis to identify key factors influencing dynamic ankle spasticity during walking in patients with stroke-induced hemiparesis and to explore objective indicators for quantifying its severity. The study yielded two main findings. First, reduced ankle plantarflexion angle and weakened dorsiflexor strength on the affected side were independently associated with the presence of walking-related dynamic spasticity. Second, prolonged double support time on the affected side was significantly correlated with the severity of dynamic spasticity during walking. These results may inform personalized rehabilitation strategies for dynamic ankle spasticity, such as assisting clinicians in determining whether antispastic interventions are needed and guiding decisions regarding the appropriate intensity or extent of such treatments.

### Lower-limb physical assessments associated with dynamic spasticity

In this study, we first examined a series of ankle physical assessment indicators based on clinical practicality and biomechanical relevance. Muscle strength assessment reflects voluntary motor function and serves as the foundation of motor control, providing essential support for joint stability and coordination during both the stance and the swing phases. Changes in muscle strength can directly affect gait stability and coordination and are closely related to the presence of spasticity [32]. Joint range of motion, particularly ankle dorsiflexion and plantarflexion angles, has also been associated with the severity of spasticity. Previous studies have shown that ankle joint angle changes during the stance phase and the swing phase are closely linked to spasticity in related muscle groups such as the gastrocnemius and soleus muscles [7, 33]. In addition, joint mobility parameters such as dorsiflexion and plantarflexion angles can help identify the presence of tendon contracture [34].

We then assessed other lower-limb physical examination findings related to dynamic spasticity. Patients with ankle spasticity often develop knee muscle tightness simultaneously. The Ely test is primarily used to evaluate the tightness or contracture of the rectus femoris muscle. It may also provide clues about abnormal quadriceps activation during gait. Studies have indicated that a positive Ely test may reflect this abnormal activation and can serve as an indirect marker of dynamic spasticity [35]. The popliteal angle is a commonly used clinical indicator to evaluate the tightness of the hamstring muscles, including the biceps femoris, semitendinosus, and semimembranosus. A larger popliteal angle often suggests increased muscle tightness and may indicate the presence of spasticity or contracture [36]. Calf circumference is an indirect indicator of muscle mass, tone, and structural adaptation. It can reflect changes in spastic muscles caused by chronic overuse or disuse and may therefore be associated with spasticity-related alterations [37].

In this study, the Model 1 results showed that, after adjustment for other variables, reduced passive ankle plantarflexion angle on the affected side and weakened dorsiflexor strength remained significantly associated with dynamic spasticity. This finding is novel and clinically important, as clinical practice typically focuses on ankle dorsiflexion in the hemiparetic limb but often overlooks ankle plantarflexion. During walking, ankle dorsiflexion and plantarflexion work in coordination to complete the gait cycle, demonstrating functional complementarity and bidirectional neural regulation [38].

After a stroke, a reduced passive ankle plantarflexion angle on the affected side is typically influenced by several interacting mechanisms, including plantarflexor spasticity or contracture, inappropriate dorsiflexor activation, impaired reciprocal inhibition, abnormal muscle co-contraction, biomechanical adaptations, and dorsiflexor weakness [7, 17]. Spasticity or shortening of the gastrocnemius–soleus complex can directly limit passive plantarflexion. During passive movement, the tibialis anterior may activate when relaxation is required, producing dorsiflexion-directed resistance that restricts the attainable angle. Impaired reciprocal inhibition and excessive co-contraction further increase joint stiffness and reduce mobility. Biomechanical compensations, such as maintaining a slightly plantarflexed position for stability, may gradually shorten the plantarflexor muscle–tendon unit. In addition, dorsiflexor weakness may induce increased plantarflexor tone as the neuromuscular system attempts to stabilize the ankle, thereby further reducing passive plantarflexion [39, 40].

### Double support time on the affected side in 3D gait analysis is associated with the severity of dynamic ankle spasticity

In the regression analysis of Model 2, we found that prolonged double support time on the affected side was the only gait parameter significantly associated with the severity of dynamic ankle spasticity. The double support phase is a critical component of the gait cycle, referring to the period during which both feet are in contact with the ground, typically accounting for approximately 20–24% of the entire gait cycle. The double support time on the affected side primarily comprises the initial double support phase (initial contact and loading response) and the terminal double support phase (pre-swing) [41].

After a stroke, particularly under the influence of spasticity, the affected lower limb often presents with increased muscle tone, joint stiffness, and impaired motor coordination, making it difficult to complete the transition between stance and swing phases promptly, thereby leading to prolonged double support time [42]. Such prolongation is widely regarded as a compensatory strategy for impaired gait stability, as patients increase the duration of bilateral foot contact to enlarge their support base and reduce the risk of postural instability and falls [43].

During the initial double support phase, normal gait requires a stable heel strike, controlled eccentric dorsiflexion, and rapid transfer of body weight onto the affected limb [44]. However, velocity-dependent overactivation of the plantarflexors induced by spasticity, together with ankle joint stiffness and restricted dorsiflexion, limits the affected limb’s ability to accept load. As a result, patients prolong the contralateral limb’s ground-contact time to maintain stability, leading to an extended initial double support period.

In the terminal double support phase, normal gait requires the plantarflexors to generate an effective push-off and to facilitate a smooth transition from stance to swing [44]. However, dynamic spasticity often prevents timely relaxation of the plantarflexors, induces abnormal co-contraction between the dorsiflexor and plantarflexor muscles, and disrupts the initiation of knee flexion. These impairments hinder effective toe-off and the initiation of the swing phase. Consequently, patients require additional time to adjust limb posture and shift their center of mass to compensate for insufficient propulsion and impaired coordination, further prolonging the terminal double support phase.

Taken together, both the initial double support phase, which reflects load acceptance, and the terminal double support phase, which reflects push-off and swing initiation, place high demands on ankle muscle tone regulation, neuromuscular coordination, and phase-transition control. The more severe the dynamic spasticity, the more pronounced the instability and delayed movement execution during these critical gait phases, leading patients to rely increasingly on “both feet on the ground” to maintain balance [45]. Therefore, the prolongation of double support time on the affected side represents not only a compensatory response to impaired gait stability but also a sensitive indicator of the extent to which dynamic ankle spasticity interferes with gait performance. The findings of this study suggest that double support time may serve as a valuable gait-based marker for quantifying the severity of dynamic spasticity, providing important guidance for clinical evaluation and targeted rehabilitation interventions.

## Limitations

This study has several limitations. First, it employed a single-center, case-control design. Although propensity score matching was used to control for certain confounding factors, residual selection bias may still exist, and causal inferences remain limited. Future studies using multicenter, prospective cohort designs are warranted to enhance the generalizability and causal interpretability of the findings. Second, we noted that the Tardieu X score demonstrated reduced variability, indicating a mild ceiling effect. While this does not undermine the main findings, the relatively small sample size may have further limited the stability and generalizability of the regression models. Larger cohorts and more rigorous analytical approaches are needed in future research to validate and strengthen the findings. Third, we did not assess cognitive function or other neurological factors that may influence gait, so future studies should include these evaluations to improve accuracy. Fourth, although this study relied on a laboratory-based VICON system to ensure high precision in joint-level kinematics, future research may benefit from adopting wearable inertial measurement units (IMUs). IMUs offer portability and the ability to assess movement in real-world environments, which is particularly valuable for capturing dynamic spasticity during everyday walking. Despite their current limitations in measuring small joint angles and susceptibility to signal drift, continued technological advancements are likely to enhance their accuracy and clinical applicability [46].

## Clinical implications

The findings of this study have several practical implications for the clinical assessment of dynamic ankle spasticity after stroke. First, the associations of restricted ankle plantarflexion and weakened dorsiflexor strength with the presence of dynamic spasticity highlight the value of incorporating these routine bedside indicators into early screening. These measures may help clinicians identify patients at risk of walking-related spasticity prior to 3D gait analysis. Second, prolonged double support time on the affected side represents a clinically accessible gait parameter that reflects the functional impact of spasticity during real walking. This parameter can be obtained from 3D gait analysis and may also be recorded using wearable sensors in daily practice, which makes it a useful marker for monitoring gait instability and potential treatment effects. Overall, combining bedside physical assessments with gait-based indicators may support earlier detection of dynamic ankle spasticity and guide more individualized rehabilitation planning.

## Conclusions

This study demonstrated that restricted plantarflexion and weakened dorsiflexor strength are associated with the presence of dynamic ankle spasticity after stroke, and that prolonged double support time reflects its severity. These findings improve our understanding of gait-related dynamic ankle spasticity and support movement-focused clinical assessment strategies for post-stroke patients.

## Supplementary Information

Below is the link to the electronic supplementary material.

Supplementary Material 1.

Supplementary Material 2.

Supplementary Material 3.

Supplementary Material 4. Fig. 1. Key phases and events of a single gait cycle.

Supplementary Material 5. Fig. 2. Significant demographic and physical assessment differences between groups.

Supplementary Material 6. Fig. 3. Significant kinematic variables differences between groups.

Supplementary Material 7. Fig. 4. Significant spatiotemporal parameters differences between groups.

## Data Availability

The data supporting the findings of this study are available from the corresponding author, Dr. Yue Wang, upon request.

## References

[1] Wissel J, Manack A, Brainin M. Toward an epidemiology of poststroke spasticity. Neurology. 2013;80(3 Suppl 2):S13–19. 23319481 10.1212/WNL.0b013e3182762448

[2] Liao LY, Xu PD, Fang XQ, Wang QH, Tao Y, Cheng H, Gao CY. Prevalence and clinical predictors of spasticity after intracerebral hemorrhage. Brain Behav 2023, 13(3). 10.1002/brb3.2906 PMC10013944 36750443

[3] Li S, Francisco GE, Rymer WZ. A New definition of poststroke spasticity and the interference of spasticity with motor recovery from acute to chronic stages. Neurorehabilit Neural Repair. 2021;35(7):601–10. 10.1177/15459683211011214 33978513

[4] Starosta M, Marek K, Redlicka J, Miller E. Extracorporeal shockwave treatment as additional therapy in patients with post-stroke spasticity of upper limb-a narrative review. J Clin Med 2024, 13(7). 10.3390/jcm13072017 PMC11012993 38610782

[5] Alamer A, Melese H, Getie K, Deme S, Tsega M, Ayhualem S, Birhanie G, Abich Y, Yitayeh Gelaw A. Effect of Ankle joint mobilization with movement on range of motion, balance and gait function in chronic stroke survivors: systematic review of randomized controlled trials. Degener Neurol Neuromuscul Dis. 2021;11:51–60. 34512072 10.2147/DNND.S317865 PMC8420562

[6] Li S. Ankle and Foot Spasticity Patterns in Chronic Stroke Survivors with Abnormal Gait. Toxins (Basel) 2020, 12(10). 10.3390/toxins12100646 PMC7600702 33036356

[7] Peeters N, Papageorgiou E, Hanssen B, De Beukelaer N, Staut L, Degelaen M, Van den Broeck C, Calders P, Feys H, Van Campenhout A et al. The short-term impact of botulinum neurotoxin-a on muscle morphology and gait in children with spastic cerebral palsy. Toxins (Basel) 2022, 14(10). 10.3390/toxins14100676 PMC9607504 36287944

[8] OuYang Z, Shen C, Wang Y. Motion analysis for the evaluation of dynamic spasticity during walking: a systematic scoping review. Mult Scler Relat Disord. 2025;94:106273. 39827537 10.1016/j.msard.2025.106273

[9] Knikou M, Murray LM. Repeated transspinal stimulation decreases soleus H-reflex excitability and restores spinal inhibition in human spinal cord injury. PLoS ONE 2019, 14(9). 10.1371/journal.pone.0223135 PMC6762874 31557238

[10] Hofstoetter US, Freundl B, Danner SM, Krenn MJ, Mayr W, Binder H, Minassian K. Transcutaneous spinal cord stimulation induces temporary attenuation of spasticity in individuals with spinal cord injury. J Neurotrauma. 2020;37(3):481–93. 31333064 10.1089/neu.2019.6588

[11] Sandler EB, Condon K, Field-Fote EC. Efficacy of transcutaneous spinal stimulation versus whole body vibration for spasticity reduction in persons with spinal cord injury. J Clin Med 2021, 10(15). 10.3390/jcm10153267 PMC8348743 34362051

[12] Romeni S, Losanno E, Emedoli D, Albano L, Agnesi F, Mandelli C, Barzaghi LR, Pompeo E, Mura C, Alemanno F, et al. High-frequency epidural electrical stimulation reduces spasticity and facilitates walking recovery in patients with spinal cord injury. Sci Transl Med. 2025;17(780):eadp9607. 39772775 10.1126/scitranslmed.adp9607

[13] Nagel SJ, Wilson S, Johnson MD, Machado A, Frizon L, Chardon MK, Reddy CG, Gillies GT, Howard MA. III: spinal cord stimulation for spasticity: historical approaches, current status, and future directions. Neuromodulation. 2017;20(4):307–21. 28370802 10.1111/ner.12591

[14] Wang J, Fan L, Sun J, Chen J, Wang Y, Ouyang Z, Yuan Z, Sun C, Jin L, Wang Y. Relationship between reticulospinal system sensitization and proprioceptive pathways in the development of dynamic spasticity (ReProDS) post-spinal cord injury: protocol for a prospective, observational cohort study. BMC Neurol 2024, 24(1). 10.1186/s12883-024-03947-y PMC11558821 39538163

[15] Bravo-Esteban E, Taylor J, Aleixandre M, Simon-Martinez C, Torricelli D, Pons JL, Gomez-Soriano J. Tibialis Anterior muscle coherence during controlled voluntary activation in patients with spinal cord injury: diagnostic potential for muscle strength, gait and spasticity. J Neuroeng Rehabil 2014, 11. 10.1186/1743-0003-11-23 PMC3973993 24594207

[16] Singh P, Joshua AM, Ganeshan S, Suresh S. Intra-rater reliability of the modified Tardieu scale to quantify spasticity in elbow flexors and ankle plantar flexors in adult stroke subjects. Ann Indian Acad Neurol. 2011;14(1):23–6. 21633610 10.4103/0972-2327.78045 PMC3098519

[17] Lamontagne A, Malouin F, Richards CL. Locomotor-specific measure of spasticity of plantarflexor muscles after stroke. Arch Phys Med Rehabil. 2001;82(12):1696–704. 11733885 10.1053/apmr.2001.26810

[18] Faccioli S, Cavalagli A, Falocci N, Mangano G, Sanfilippo I, Sassi S. Gait analysis patterns and rehabilitative interventions to improve gait in persons with hereditary spastic paraplegia: a systematic review and meta-analysis. Front Neurol. 2023;14:1256392. 37799279 10.3389/fneur.2023.1256392 PMC10548139

[19] Munari D, Serina A, Disaro J, Modenese A, Filippetti M, Gandolfi M, Smania N, Picelli A. Combined effects of backward treadmill training and botulinum toxin type A therapy on gait and balance in patients with chronic stroke: a pilot, single-blind, randomized controlled trial. NeuroRehabilitation. 2020;46(4):519–28. 32508341 10.3233/NRE-203067

[20] Kim SK, Rha DW, Park ES. Botulinum toxin type a injections impact hamstring muscles and gait parameters in children with flexed knee gait. Toxins 2020, 12(3). 10.3390/toxins12030145 PMC7150820 32120947

[21] Li F, Wu Y, Li X. Test-retest reliability and inter-rater reliability of the Modified Tardieu Scale and the Modified Ashworth Scale in hemiplegic patients with stroke. Eur J Phys Rehabil Med. 2014;50(1):9–15. 24309501

[22] Logigian MK, Samuels MA, Falconer J, Zagar R. Clinical exercise trial for stroke patients. Arch Phys Med Rehabil. 1983;64(8):364–7. 6882175

[23] Muhlenhaupt M, Measurement of joint motion, - a guide to. goniometry, 1st edition - norkin,cc, white,DJ. Am J Occup Ther. 1986;40(5):369–70.

[24] Gajdosik R, Lusin G. Hamstring muscle tightness. Reliability of an active-knee-extension test. Phys Ther. 1983;63(7):1085–90. 6867117 10.1093/ptj/63.7.1085

[25] Marks MC, Alexander J, Sutherland DH, Chambers HG. Clinical utility of the Duncan-Ely test for rectus femoris dysfunction during the swing phase of gait. Dev Med Child Neurol. 2003;45(11):763–8. 14580132 10.1017/s0012162203001415

[26] Wu G, Siegler S, Allard P, Kirtley C, Leardini A, Rosenbaum D, Whittle M, D’Lima DD, Cristofolini L, Witte H, et al. ISB recommendation on definitions of joint coordinate system of various joints for the reporting of human joint motion–part I: ankle, hip, and spine. International Society of Biomechanics. J Biomech. 2002;35(4):543–8. 11934426 10.1016/s0021-9290(01)00222-6

[27] Fukuchi CA, Fukuchi RK, Duarte M. Effects of walking speed on gait biomechanics in healthy participants: a systematic review and meta-analysis. Syst Rev. 2019;8(1):153. 31248456 10.1186/s13643-019-1063-z PMC6595586

[28] Kiss RM, Kocsis L, Knoll Z. Joint kinematics and spatial–temporal parameters of gait measured by an ultrasound-based system. Med Eng Phys. 2004;26(7):611–20. 15271289 10.1016/j.medengphy.2004.04.002

[29] Herssens N, Verbecque E, Hallemans A, Vereeck L, Van Rompaey V, Saeys W. Do spatiotemporal parameters and gait variability differ across the lifespan of healthy adults? A systematic review. Gait Posture. 2018;64:181–90. 29929161 10.1016/j.gaitpost.2018.06.012

[30] Eltoukhy M, Oh J, Kuenze C, Signorile J. Improved kinect-based spatiotemporal and kinematic treadmill gait assessment. Gait Posture. 2017;51:77–83. 27721202 10.1016/j.gaitpost.2016.10.001

[31] Perry JBJ. Gait Analysis: Normal and Pathological Function. 2nd ed. edn. Boca Raton: CRC; 2010.

[32] Sunnerhagen KS, Opheim A, Alt Murphy M. Onset, time course and prediction of spasticity after stroke or traumatic brain injury. Ann Phys Rehabil Med. 2019;62(6):431–4. 29753889 10.1016/j.rehab.2018.04.004

[33] Liu H, Fan L, Li J, Dangol S, Talifu Z, Ma X, Gong H, Du L. Combined selective peripheral neurotomy in the treatment of spastic lower limbs of spinal cord injury patients. Acta Neurochir (Wien). 2022;164(8):2263–9. 35665860 10.1007/s00701-022-05265-z PMC9166246

[34] Gatt A, Chockalingam N. Clinical assessment of ankle joint dorsiflexion: a review of measurement techniques. J Am Podiatr Med Assoc. 2011;101(1):59–69. 21242472 10.7547/1010059

[35] Lee SY, Sung KH, Chung CY, Lee KM, Kwon SS, Kim TG, Lee SH, Lee IH, Park MS. Reliability and validity of the Duncan-Ely test for assessing rectus femoris spasticity in patients with cerebral palsy. Dev Med Child Neurol. 2015;57(10):963–8. 25846806 10.1111/dmcn.12761

[36] Hajibozorgi M, Hijmans JM, Greve C. The functional popliteal angle test can detect features of hamstring spasticity. Clin Biomech (Bristol). 2025;125:106523. 40245565 10.1016/j.clinbiomech.2025.106523

[37] Picelli A, Tamburin S, Cavazza S, Scampoli C, Manca M, Cosma M, Berto G, Vallies G, Roncari L, Melotti C, et al. Relationship between ultrasonographic, electromyographic, and clinical parameters in adult stroke patients with spastic equinus: an observational study. Arch Phys Med Rehabil. 2014;95(8):1564–70. 24792138 10.1016/j.apmr.2014.04.011

[38] Young DR, Banks CL, McGuirk TE, Patten C. Evidence for shared neural information between muscle synergies and corticospinal efficacy. Sci Rep 2022, 12(1). 10.1038/s41598-022-12225-1 PMC9142531 35624121

[39] Brough LG, Kautz SA, Neptune RR. Muscle contributions to pre-swing biomechanical tasks influence swing leg mechanics in individuals post-stroke during walking. J Neuroeng Rehabil 2022, 19(1). 10.1186/s12984-022-01029-z PMC9166530 35659252

[40] Kitatani R, Ohata K, Aga Y, Mashima Y, Hashiguchi Y, Wakida M, Maeda A, Yamada S. Descending neural drives to ankle muscles during gait and their relationships with clinical functions in patients after stroke. Clin Neurophysiol. 2016;127(2):1512–20. 26601960 10.1016/j.clinph.2015.10.043

[41] Perry J, Burnfield JM. Gait analysis. Normal and pathological function 2nd ed. California: Slack 2010.

[42] Srivastava S, Patten C, Kautz SA. Altered muscle activation patterns (AMAP): an analytical tool to compare muscle activity patterns of hemiparetic gait with a normative profile. J Neuroeng Rehabil. 2019;16(1):21. 30704483 10.1186/s12984-019-0487-y PMC6357420

[43] Mizuta N, Hasui N, Higa Y, Matsunaga A, Ohnishi S, Sato Y, Nakatani T, Taguchi J, Morioka S. Identifying impairments and compensatory strategies for temporal gait asymmetry in post-stroke persons. Sci Rep. 2025;15(1):2704. 39838057 10.1038/s41598-025-86167-9 PMC11751082

[44] Webster JB, Darter BJ. Principles of normal and pathologic gait. Atlas of Orthoses and Assistive Devices. edn.: Elsevier; 2026. pp. 54–68. e51.

[45] Li Y, Lyu Y, Song R. Phase-dependent modulation of muscle activity and intermuscular coupling during walking in patients after stroke. IEEE Trans Neural Syst Rehabil Eng. 2023;31:1119–27. 37022070 10.1109/TNSRE.2023.3238758

[46] de l’Escalopier N, Voisard C, Jung S, Michaud M, Moreau A, Vayatis N, Denormandie P, Verrando A, Verdaguer C, Moussu A, et al. Inertial measurement units to evaluate the efficacity of Equino Varus Foot surgery in post stroke hemiparetic patients: a feasibility study. J Neuroeng Rehabil. 2024;21(1):182. 39407309 10.1186/s12984-024-01469-9 PMC11481626

