# Supplementary material for: Three-dimensional gait analysis for assessing dynamic ankle spasticity after stroke
Source: J Neuroeng Rehabil. 2026 Apr 2;23:159. doi: 10.1186/s12984-026-01968-x (PMC13169611; doi:10.1186/s12984-026-01968-x)
Supplement: Supplementary file 1 — Supplementary Material 1. [file 12984_2026_1968_MOESM1_ESM.docx]

***Table SM 1*** *Correlation between disease duration and physical assessment factors with Tardieu Y and X values in the spasticity group*

|  | r_s_-Y | *p*-value | r_s_-X | *p*-value |
| --- | --- | --- | --- | --- |
| Duration (months) | -0.02 | 0.87 | 0.02 | 0.91 |
| Popliteal angle (affected, ˚) | -0.13 | 0.40 | -0.13 | 0.43 |
| Dorsiflexion angle (unaffected, ˚) | 0.04 | 0.82 | 0.06 | 0.71 |
| Dorsiflexion angle (affected, ˚) | 0.11 | 0.49 | -0.34 | 0.03* |
| Plantarflexion angle (affected, ˚) | 0.12 | 0.46 | 0.26 | 0.09 |
| Dorsiflexor strength (affected) | 0.07 | 0.68 | -0.37 | 0.02* |
| Plantarflexor strength (affected) | 0.11 | 0.47 | -0.43 | 0.005** |

**p* < 0.05, ***p* < 0.01. r_s_: Spearman correlation coefficient.
