# Supplementary material for: Three-dimensional gait analysis for assessing dynamic ankle spasticity after stroke
Source: J Neuroeng Rehabil. 2026 Apr 2;23:159. doi: 10.1186/s12984-026-01968-x (PMC13169611; doi:10.1186/s12984-026-01968-x)
Supplement: Supplementary file 2 — Supplementary Material 2. [file 12984_2026_1968_MOESM2_ESM.docx]

*Table SM 2* *Correlation of kinematic and spatiotemporal parameters with Tardieu Y and X values in the spasticity group*

|  | r_s_ -Y | *p*-value | r_s_ -X | *p*-value |
| --- | --- | --- | --- | --- |
| Initial contact ankle angle (unaffected, °) | 0.33 | 0.04* | -1.93 | 0.22 |
| Initial contact ankle angle (affected, °) | 0.24 | 0.12 | -0.31 | 0.045* |
| Max dorsiflexion angle (affected, °) | 0.15 | 0.33 | -0.08 | 0.62 |
| Speed (cm/s) | 0.07 | 0.64 | -0.26 | 0.10 |
| Cadence (affected, steps/min) | 0.12 | 0.44 | -0.28 | 0.07 |
| Step width (cm) | 0.16 | 0.32 | -0.32 | 0.04* |
| Step length (unaffected, cm) | 0.15 | 0.36 | -0.002 | 0.99 |
| Step length (affected, cm) | -0.16 | 0.31 | -0.10 | 0.51 |
| Step length (unaffected, % height) | 0.14 | 0.36 | 0.02 | 0.91 |
| Step length (affected, % height) | -0.16 | 0.31 | -0.06 | 0.72 |
| Stance phase (unaffected, s) | -0.07 | 0.68 | 0.37 | 0.02* |
| Stance phase (affected, s) | -0.08 | 0.62 | 0.38 | 0.01* |
| Stance phase (% cycle, unaffected) | 0.04 | 0.80 | 0.24 | 0.12 |
| Double support (unaffected, s) | -0.02 | 0.89 | 0.30 | 0.06 |
| Double support (affected, s) | 0.05 | 0.76 | 0.44 | 0.004** |
| Double support (% cycle, unaffected) | 0.06 | 0.71 | 0.16 | 0.30 |
| Swing phase (affected, s) | -0.34 | 0.03* | 0.32 | 0.04* |
| Swing phase (% cycle, unaffected) | -0.04 | 0.80 | -0.24 | 0.12 |
| Gait cycle duration (unaffected, s) | -0.11 | 0.47 | 0.38 | 0.02* |
| Gait cycle duration (affected, s) | -0.12 | 0.47 | 0.39 | 0.01* |

**p* < 0.05, ***p* < 0.01. r_s_: Spearman correlation coefficient.
