# Supplementary material for: Three-dimensional gait analysis for assessing dynamic ankle spasticity after stroke
Source: J Neuroeng Rehabil. 2026 Apr 2;23:159. doi: 10.1186/s12984-026-01968-x (PMC13169611; doi:10.1186/s12984-026-01968-x)
Supplement: Supplementary file 3 — Supplementary Material 3. [file 12984_2026_1968_MOESM3_ESM.docx]

*Table SM 3 Collinearity check: correlation of kinematic and spatiotemporal parameters with double support time (Affected Side) in the spasticity group*

|  | r_s_ -Double support (affected, s) | *p*-value |
| --- | --- | --- |
| Initial contact ankle angle (affected, °) | -0.09 | 0.57 |
| Step width (cm) | -0.12 | 0.45 |
| Stance phase (unaffected, s) | 0.88 | <0.001*** |
| Stance phase (affected, s) | 0.89 | <0.001*** |
| Swing phase (affected, s) | 0.36 | 0.02* |
| Gait cycle duration (unaffected, s) | 0.87 | <0.001*** |
| Gait cycle duration (affected, s) | 0.88 | <0.001*** |

**p* < 0.05, ***p* < 0.01, ****p* < 0.001. r_s_: Spearman correlation coefficient.
