# Supplementary figures and images for: Three-dimensional gait analysis for assessing dynamic ankle spasticity after stroke
Source: J Neuroeng Rehabil. 2026 Apr 2;23:159. doi: 10.1186/s12984-026-01968-x (PMC13169611; doi:10.1186/s12984-026-01968-x)

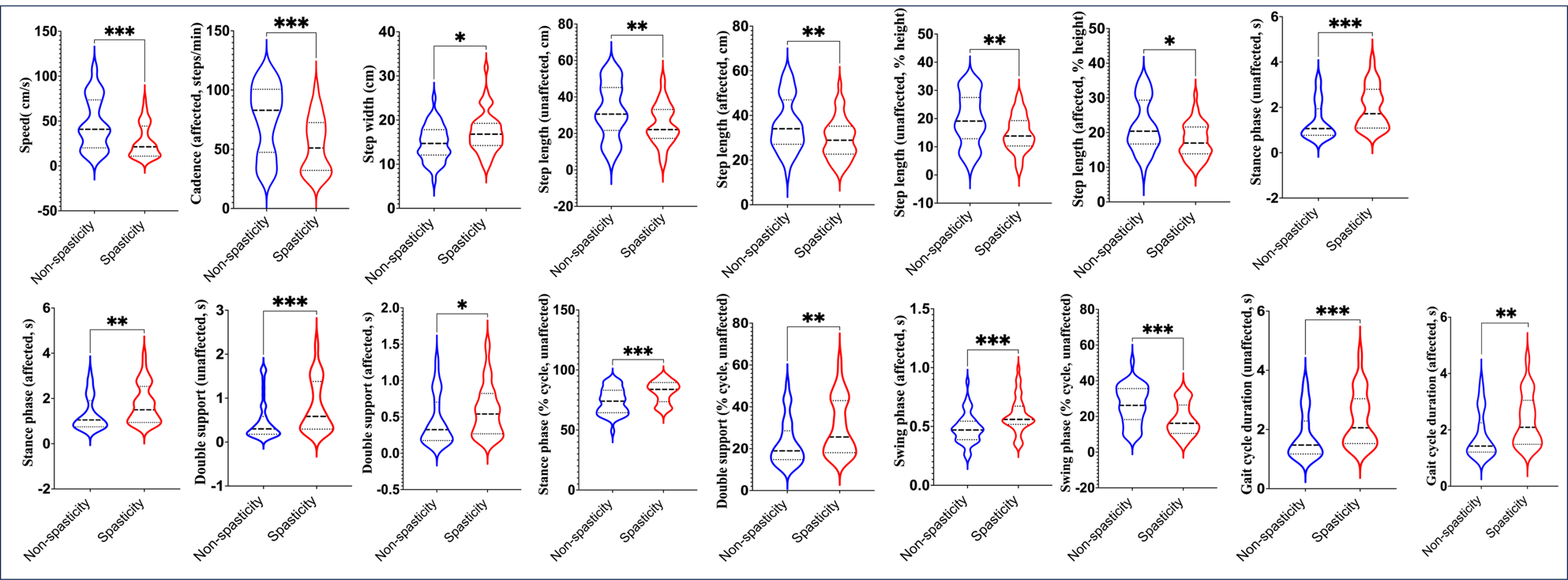

Supplement: Supplementary file 4 — Supplementary Material 4. Fig. 1. Key phases and events of a single gait cycle. [file 12984_2026_1968_MOESM4_ESM.tif]

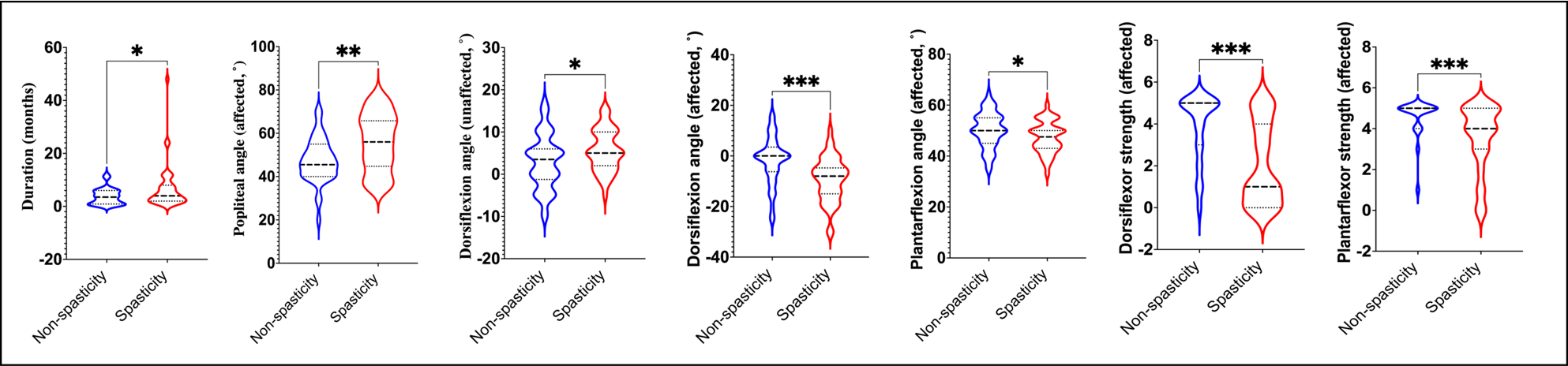

Supplement: Supplementary file 5 — Supplementary Material 5. Fig. 2. Significant demographic and physical assessment differences between groups. [file 12984_2026_1968_MOESM5_ESM.tif]

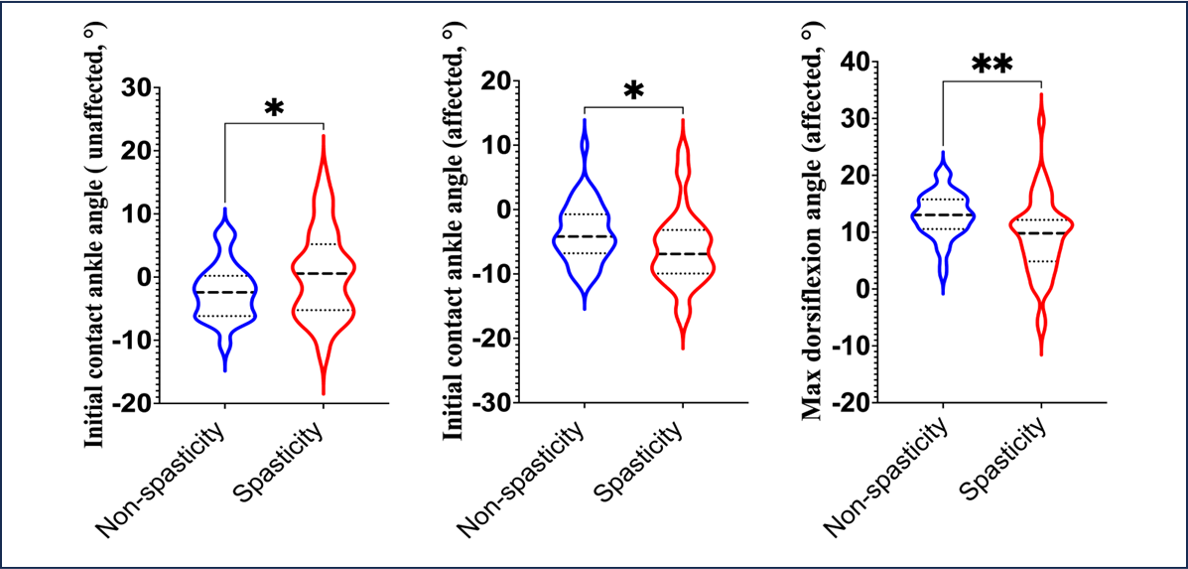

Supplement: Supplementary file 6 — Supplementary Material 6. Fig. 3. Significant kinematic variables differences between groups. [file 12984_2026_1968_MOESM6_ESM.tif]

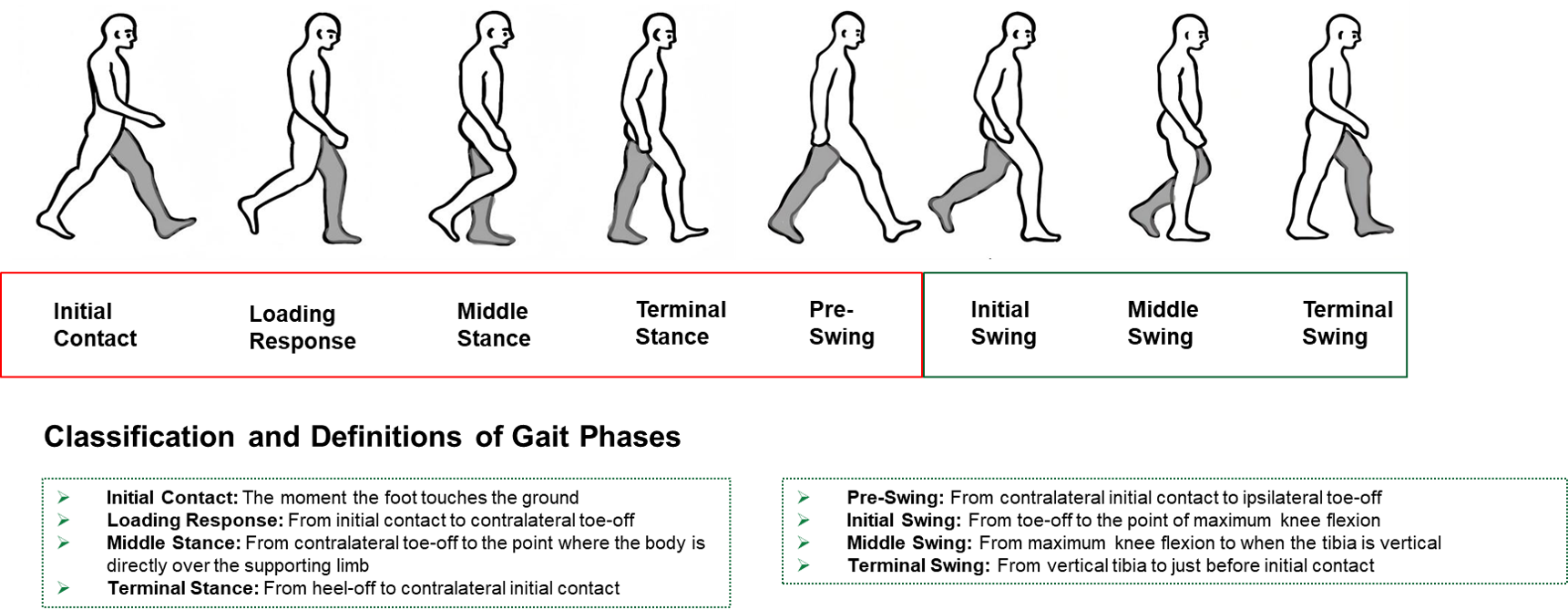

Supplement: Supplementary file 7 — Supplementary Material 7. Fig. 4. Significant spatiotemporal parameters differences between groups. [file 12984_2026_1968_MOESM7_ESM.tif]
